# A Case Report of Intraductal Papillary-Mucinous Neoplasm of the Pancreas Showing Morphologic Transformation during Followup Periods

**DOI:** 10.1155/2009/373465

**Published:** 2009-10-15

**Authors:** Yuichi Sanada, Shinji Osada, Yoshihiro Tanaka, Yasuharu Tokuyama, Kazuhiro Yoshida

**Affiliations:** Department of Surgical Oncology, Gifu Graduate School of Medicine, 1-1 Yanagido, Gifu city 501-1194, Japan

## Abstract

A 64-year-old man underwent MRCP for further examination of gallbladder stones and IPMN of branch-type (IPMN-Br) was pointed out. Yearly MRCP had revealed the gradual increase of the cystic components, marked dilation of the main pancreatic duct (MPD), and filling defects in the MPD. After follow-up for three years, he underwent pancreatoduodenectomy. Histologically, the dilated MPD and connecting dilated branch ducts were filled with nodular growth of tumor cells consisting of gastric-type adenoma with pyloric gland-like structures. In the MPD, a transition from gastric-type adenoma to intestinal-type carcinoma was observed. In addition, in a dilated branch duct, some components of intestinal-type carcinoma with marked arborizing structures were observed. A minimally invasion was observed around branch ducts. Immunohistochemistry revealed diffuse nuclear accumulation of PCNA and Ki67 in the tumor cells of branch dusts. Our observations suggest that the secondary infiltration to the MPD of IPMN-Br and IPMN-Br possesses malignant potential for microinvasion.

## 1. Introduction

 The malignant potential of intraductal papillary-mucinous neoplasm (IPMN) of the pancreas has been estimated based on three viewpoints: histologic grades (adenoma, carcinoma and their borderline lesion), localization (main duct-type and branch duct-type), and histologic subtypes (gastric type, intestinal type, pancreatobiliary type, and oncocytic type) [1, 2]. These viewpoints have a deep connection for each other, such as, the gastric type, which occurs in the periphery of the pancreatic parenchyma, corresponds to the branch duct type, and which usually shows a multicystic lesion with less invasive pattern, adenoma [2]. Then, branch-type IPMN (IPMN-Br) has been usually an object for just follow up. However, recent guidelines indicated operative indication as the followings: symptoms attributable to the cyst, dilation of the main pancreatic duct (MPD) over 10 mm, cyst size over 30 mm with the intramural nodule, or cystic fluid with cytological malignancy [3]. In fact, relative surgical indications were applied for cases with disease development such as pancreatitis, positive cytology, or solid mass formation in the followup term for IPMN-Br [4, 5]. By contrast, there was no report describing the IPMN-Br to show morphologic change and transformation to the main duct. In the present, our experienced IPMN-Br case, which is visualized for transition gradually from branch to MPD in three years followup images, was found to include several patterns of subtypes by histopathological study. This phenomenon might be critical to estimate the malignant potential or surgical indication for IPMN-Br.

## 2. Case Report

**Figure 1:**
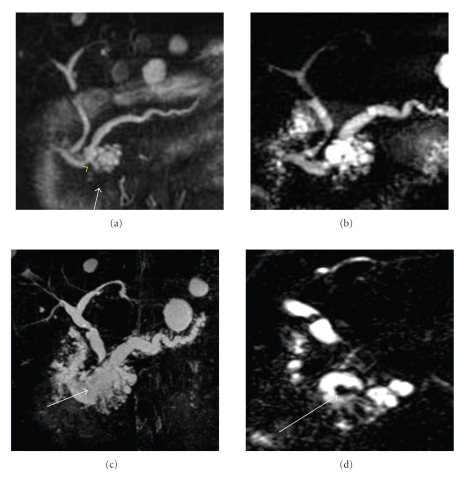
(a) MRCP in 2003. Cystic dilation of branch ducts is observed in the pancreas head (arrow). A duct-cyst junction is visible (yellow arrowhead). (b) MRCP in 2004 shows the increasing of size and dilation of the main pancreatic duct. (c) MRCP in 2005. The boundary between the main pancreatic duct and the cystic components of branch ducts disappears. (d) MRCP in 2006. Filling defects in the main pancreatic duct are visible (arrow).

A continuous image of 64-year-old man, who was referred to the Department of Medicine at our hospital for further evaluation of gallbladder stones, was shown in Figure 1. A magnetic resonance cholangiopancreatography (MRCP) showed a multilocular cystic lesion for 20 mm in diameter in the head of the pancreas. The main pancreatic duct was noted smoothly except for the head adjacent to the multilocular cyst. Although these findings suggested a cystic neoplasm such as serous cystadenoma, the existence of duct-cyst connection (arrow) strongly suggested that the most likely diagnosis was intraductal papillary-mucinous neoplasm (IPMN) mainly located in the branch ducts. Followup MRCP revealed gradual increase of the cystic components and its sizes, from 2.0 cm to 3.8 cm with dilation of MPD over 10 mm until 2005. In 2006, since intraductal nodule with elevation of serum amylase appeared, surgical indication was evaluated. The levels of serum tumor marker, such as carcinoembryonic antigen (CEA) or carcinohydrate antigen 19-9 (CA19-9), were in normal range. The cytological study for pancreatic juice showed findings for high-grade intraductal papillary-mucinous carcinoma (IPMC); therefore, surgical procedure was selected. On laparotomy, a cystic mass, 4.0 cm in diameter, was identified in the pancreatic head. Distal pancreatic parenchyma showed marked fibrosis. Pancreatoduodenectomy with regional lymph nodes dissection was performed. Transection of the pancreas was performed on the neck of the pancreas. Intraoperative US revealed no additional cystic mass in the residual pancreas. 

**Figure 2:**
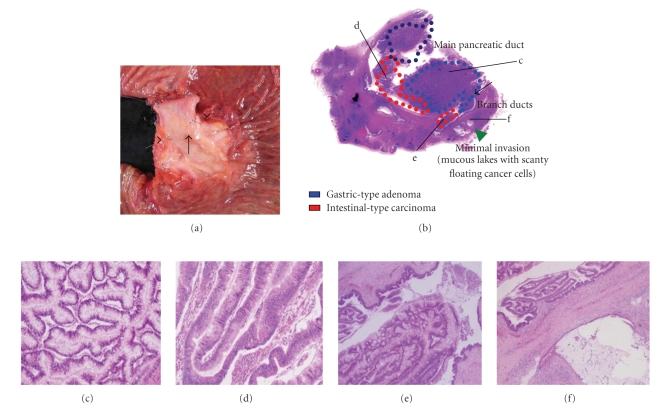
(a) Resected specimens show marked dilation of the main pancreatic duct and papillary tumor (arrow) with cystic dilation of branch ducts (arrowhead). (b) Representative section and histologic distribution of the tumor. The boundary between the main pancreatic duct and a branch duct is visible (arrow). (c) Gastric-type adenoma is made up of columunar cells with basally-oriented nuclei, showing closely packed glands with anastomosing pattern (d) Intestinal-type carcinoma shows marked pseudostratification and nuclear atypia showing villous configuration (e) In a branch duct, intestinal-type carcinoma components with arborizing features are observed (f) A minimally invasive lesion is observed adjacent to branch ducts. Floating tumor cells in the mucous lake is visible (I, arrow).

**Figure 3:**
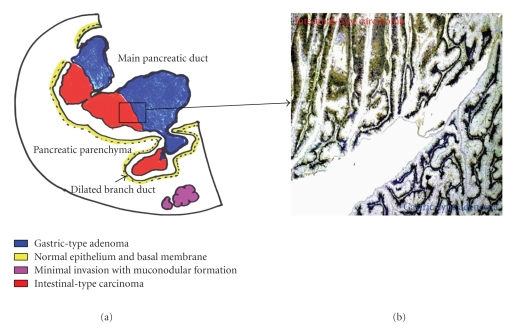
A schematic presentation of histologic subtypes in the present case (a). An immunohistochemical image for MUC2 at the boundary between gastric-type adenoma and intestinal-type carcinoma. Only the intestinal-type carcinoma shows diffuse staining for MUC2 (b).

The standard study for resected specimen was described in Figure 2. Macroscopically, cystic dilation of MPD was filled with soft papillary tumor producing mucin. And solid tumor in MPD and communicating branch ducts was visualized with different components: gastric-type adenoma showing closely packed tubular glands similar as pyloric gland-like morphology and dark eosinophilic cells with marked pseudostratificatied spindle nuclei for an intestinal-type carcinoma. In branch ducts, components of intestinal-type carcinoma showed irregular arborizing growth. Adjacent to the branch duct, a minimal invasion was observed as a dilated mucouslake containing scanty cancer cells floating in the abundant mucin, indicating mucinous carcinoma. Under consideration for the invasive components existed with the distance of less than 5 mm [6], this growth pattern was corresponded to minimal invasion. Each subtype was confirmed by mucin phenotype (Figure 3, right column). Schematic presentation of histologic distribution in the present case is shown in Figure 3 (left column). 

**Figure 4:**
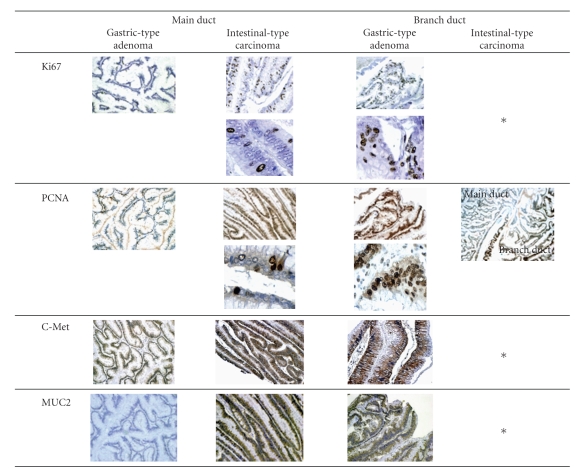
Representative immunohistochemical images in several components of the present tumor.

To estimate the malignant potential, additional immunohistochemistry (IHC) was performed by using primary antibodies as followed: Ki67 (clone MIB-1, Nichirei, 1:200), proliferating cell nuclear antigen (clone PCNA, DakoCytomation, 1:200), and c-Met (clone c-Met, Santa Cruz Biotechnology Inc, 1:200), as described in previous report. These molecules have been reported to be involved in the early events of carcinogenesis, formation of in situ carcinoma in the stepwise progression of IPMN and ordinary pancreatic ductal carcinoma. The expression of the three molecules was independently evaluated by two investigators (Y S and S K). Evaluation of the staining was determined based on the following criteria. In Ki67 and PCNA, stained cells were accounted for less than 10% regarding for negative (0), and 10% or more for positive with further classified 10% to 30% (1+) and over 30% (2+). For c-Met, positivity was rated for 1+ or 2+ under 30% cut-off line, respectively. Representative images and results for IHC are shown in Figure 4 and Table 1. Ki67 and PCNA were focally stained in the intestinal-type carcinoma in the MPD. In branch ducts, intestinal-type carcinoma components showed diffuse staining for Ki67 and PCNA. In addition, PCNA was focally stained in gastric-type adenoma components in branch ducts, but not in the MPD. 

C-Met was diffusely expressed in the intestinal-type carcinoma, whereas gastric-type adenoma components showed focal and thin staining both in the MPD and branch ducts. 

The microscopic and immunohistochemical findings indicated a diagnosis of combined type IPMC with minimal invasion from branch duct. The pancreatic parenchyma showed marked fibrotic change. No lymph node metastasis was identified. Although the nodular growth of IPMN cells was observed adjacent to the resection margin of the MPD, the epithelium of the MPD at the resection margin was lined with normal epithelial cells. He is in good condition without any recurrence sign for these 26 months. 

## 3. Discussion

In common, the indication of surgical procedures for IPMN-Br was accepted for the presence of symptom, solid mass formation, or positive cytology, suggesting the mucinous cystic neoplasm. Otherwise, followup regimens based on clinical experiences have no strong evidence, according to the past report, once a year evaluation for lesions fewer than 10 mm, 6 to 12 months for 10 to 20 mm, and 3 to 6 months for over 20 mm [7]. Despite IPMN-Br was known for less invasive comparing to the main-duct type, its malignant morphology was also demonstrated [4]. Taken together, the malignant potential is developing up to the size. In fact, carcinoma in situ or invasive carcinoma was detected in 22% of resected IPMN-Br [5]. In the present case, the cystic mass grew up gradually year by year and the size was 3.8 mm at the time of operation after 36 months followup. According to the past report for 66 conservative cases, 11 IPMN-Br were decided for surgical procedure after a median follow-up of 41 months [4]; 9 of the 11 patients had malignancy, including 4 carcinoma in situ and 5 invasive carcinomas. 

Waters et al. enhanced the critical role of MRCP for followup to provide the favorite information of IPMN with matching actual pathology [8]. In the present case also, the connection of cystic components to the MPD was clearly detected due to MRCP, and the cystic lesion was found to change its feature to grow up to the MPD. However, among our referring to the past reports, the gradual infiltration from IPMN-Br to the MPD during follow-up has not been described. The present case was found to have two independent subtypes, gastric-type and intestinal-type, supporting the recognition for IPMN to compose a combination of over two subtypes [1]. Previously, we also described another case of IPMN with gastric-type adenoma and intestinal-type borderline lesion [9]. The gastric-type adenoma of the IPMN is known to be localized just in the peripheral branches without any malignant potential [10, 11], supporting that the IPMN-Br has to be applied for followup. Then, the first MRCP offered clinical imaging course demonstrated no doubt for the present IPMN to rise from the peripheral branch. On the other hand, in the resected specimen, both gastric-type adenoma and intestinal-type carcinoma were detected in both the MPD and branch ducts, demonstrating the possibility for IPMN-Br to change the malignant morphology. The present feature is usually not typical for ordinary gastric-type adenoma [12], and the distribution or histologic features in branch ducts of the present case differ from generally believed consensus in terms of the following viewpoints. First, some papillary components in branch ducts included intestinal carcinoma. Second, minimally invasion was observed adjacent to a branch duct, not to the MPD. Findings in the present case suggest that tumor cells in branch ducts possess malignant potential leading carcinogenesis. 

Recently Ban et al. reported a case of gastric-typed IPMN-Br with focal nodular growth to progress intraductal carcinoma of pancreatobiliary type [13]. The present case might be included these rare situation. 

Imaging features during follow-up support the idea that intraductal tumor cells in the MPD can be recognized as a later stage in the present case, harboring more malignant potential than those in branch ducts. Intestinal-type carcinoma in the MPD is derived from the continuous progression that corresponds to the branch duct type showing low histologic grade (adenoma). 

To evaluate the further actions for malignant potential in IPMN-Br, IHC study was selected additionally. 

Ki67 and PCNA were found to show positivity ratio increasing with increasing grade of atypism in IPMN [14, 15]. In the present case, the most diffuse staining positively of Ki67 and PCNA was observed in the components of intestinal-type carcinoma of the present IPMN in branch duct, but not in the MPD (Table 1). 

Taken together, the present tumor had intestinal type with high-grade malignant potential in early stage. PCNA was also indicated to promote the proliferation of pancreatic ductal carcinoma with interaction of KIAA0101 and Ki67 and then play a critical role in the regulation of cell cycle [16]. These results suggest that tumor components with malignant potential in peripheral branch ducts develop the proliferation and formation of invasive potency. 

In addition, c-Met is well known as a receptor of hepatocyte growth factor and is also associated with early events of carcinogenesis in the pancreas [17]. And, the high expression of c-Met was also found to involve in intestinal metaplasia of the bile duct epithelium, meaning its relation to the intestinal pathway during carcinogenesis [17]. Then, c-Met expression was most diffusely noted in intestinal type carcinoma regardless of its localization (the MPD or branch ducts), suggesting that the intestinal pathway is associated with the morphogenesis of IPMN progression in branch ducts. 

In conclusion, the present clinical, regular histological, and immunohistochemical observations provided evidence that the components in branch ducts also might possess malignant potential leading invasive carcinoma. Further IHC study to reveal the malignant potential will lead the extend strategy in the future.

## Figures and Tables

**Table 1:** Immunohistyochemical results in several components.

Protein	Main G-ad	Int-ca	Branch G-ad	Int-ca
Ki67	0	1+	0	2+
PCNA	0	1+	1+	2+
c-Met	1+	2+	0+	2+

G-ad: gastric-type adenoma; Int-ca: intestinal-type carcinoma.
